# Glycine protects against doxorubicin-induced heart toxicity in mice

**DOI:** 10.1007/s00726-023-03261-w

**Published:** 2023-03-26

**Authors:** Mayada I. Shosha, Fawzia Z. El-Ablack, Entsar A. Saad

**Affiliations:** Chemistry Department, Faculty of Science, Damietta University, Damietta, 34517 Egypt

**Keywords:** Heart diseases, Anti-oxidative, Oxidative stress, Pro-inflammatory, Anti-inflammatory, Immunity

## Abstract

Doxorubicin (DOXO) is a well-known cancer chemotherapeutic. However, its toxic effect on the heart limits its clinical application. This study aimed to assess the effectiveness of glycine administration to counteract the DOXO-induction of cardiomyopathy in mice. Fifty male albino mice were divided into five groups (*n* = 10/group) as follows: control, DOXO, Gp100, Gp150, and Gp200. Histopathological examination of the heart, and biochemical examinations for heart function (creatine phosphokinase (CPK), lactate dehydrogenase (LDH), and aspartate aminotransferase (AST)), inflammation (tumor necrosis factor-alpha (TNF-α) and interleukin 10 (IL-10)), oxidative stress (malondialdehyde (MDA), glutathione (GSH), superoxide dismutase (SOD), catalase, nitric oxide (NO), and uric acid), kidney function (urea and creatinine), and minerals (calcium, phosphorus, sodium, and potassium) were carried out. Cardiomyopathy induced by DOXO treatment (15 mg/kg total dose) was ascertained via pathological alterations seen in heart tissue and verified biochemically via increases (*P* < 0.001) in CPK, LDH, AST, TNF-α, IL-10, MDA, NO, Na, and K levels along with decreases (*P* < 0.001) in GSH, SOD, catalase, and uric acid. Glycine co-treatment, using doses of 100, 150, and 200 mg/kg, in a dose-dependent manner, displayed ameliorated heart architecture, significantly (*P* < 0.001) improved biochemical heart function tests, reduced oxidative stress and inflammation, and controlled mineral levels. The positive actions of glycine in DOXO-induced cardiotoxicity amelioration via modulating oxidative stress, inflammation, and immunity are confirmed. Glycine antioxidative properties may be behind its positive outcomes. Finally, we present glycine as a worthy possible option against DOXO-induced heart damage after more validation.

## Introduction

Heart diseases are the top cause of death worldwide, followed by cancer. In 2019, heart diseases were the cause of 17.9 million deaths (32% of all world deaths); above three-quarters of these deaths were connected to low- and middle-income nations (WHO 2021).

Heart toxicity is a well-noted negative outcome of anthracyclines, including doxorubicin. This anthracyclines-induced cardiomyopathy limits their clinical use (Cardinale et al. 2020). Cardiomyopathies are a diverse batch of myocardium diseases marked by structural/or functional irregularities which badly affect blood pumping. They may extend to involve blood outflow hindrance during the cardiac cycle (Jarvis and Saman 2018). Cardiomyopathies frequently drive to cardiovascular death or advanced heart failure-related dysfunction (Jarvis 2019).

Doxorubicin (DOXO) drug (trade names: Adriamycin, Rubex) is one of the anthracyclines antibiotics. Naturally, it is produced by the fungus *Streptococcus peucetius var. caesius*. It has antitumor activity and, currently, is clinically used as a chemotherapeutic drug for many types of solid tumors. Unfortunately, its chemotherapeutic use is confined due to severe side effects, including cardiomyopathy, nephrotoxicity, hepatotoxicity, and neurotoxicity (Kalyanaraman 2020). DOXO-induced cardiomyopathy can lead to heart failure (Roberts et al. 2018), a potentially fatal condition (Metra and Teerlink 2017). Heart failure occurs in about 3–5% with a DOXO cumulative dose of 400 mg/m^2^ (Zamorano et al. 2016). DOXO-cardiomyopathy pathogenesis has been extensively studied and has become relatively understood. Yet, no successful therapy for DOXO-cardiomyopathy is present (Chatterjee et al. 2010). Therefore, searching for an effective cure is mandatory.

Amino acid glycine, composed of a carbon atom attached to an amino group, a carboxylic acid group, and two hydrogens, is the simplest among amino acids. Our body can synthesize glycine, so it is considered non-essential (El Hafidi et al. 2006; Zhong et al. 2012). Chiefly, it is a neutral and metabolically passive amino acid (Pérez-Torres et al. 2017). At the biosynthesis level, it is a substrate for numerous vital biomolecules’ synthesis, e.g., proteins, porphyrins, glucose, creatinine, glutathione (GSH), neurotransmitters, bile salts, and purine nucleotides. Besides, as a part of GSH, it participates in antioxidant defense and detoxification reactions (Xie et al. 2016). Glycine has anti-inflammatory, cytoprotective, and immuno-modulatory effects. It remarkably lowers the lipopolysaccharide-induced release of superoxide ions and tumor necrosis factor-alpha (TNF-α) (Pérez-Torres et al. 2017). Glycine cardio-protective effects have been reported (Li et al. 2015) in vivo on cardiovascular diseases caused by myocardial ischemia (Zhong et al. 2012), hypertension (El Hafidi et al. 2006), or by hyperglycemia (Li et al. 2015). Also, pre-treatment with glycine ameliorated the pressure overload-induced murine cardiac hypertrophy, and knocking down glycine receptor-α2 in rats exhibited canceling the protective effect of glycine on the myocardium. Moreover, in culture experiments, glycine seemed to antagonize angiotensin II-activated flow of transforming growth factor beta (TGF-β) and endothelin-1 by cardiac cells that prohibited an excessive production of collagens in rat fibroblasts (Lu et al. 2017). It also has been inversely correlated with acute myocardial infarction risk in patients with suspected angina pectoris (Ding et al. 2015). In addition, it reduced platelet aggregation and ameliorated microcirculation (Ruiz-Ramirez et al. 2014). Among many modes of action, which have been proposed for glycine cytoprotective influence, decreasing cellular membrane permeability, decreasing apoptosis (Zhong et al. 2012), decreasing free radicals production (El Hafidi et al. 2006), and GSH synthesis motivation (Pérez-Torres et al. 2017) were involved.

As DOXO cardiotoxicity is a condition that involves increased oxidative stress (Chatterjee et al. 2010) and glycine has been established to protect against oxidative stress in many pathological conditions (El Hafidi et al. 2006), this study was designed to investigate the possible protective effect of glycine treatment on cardiac toxicity induced by DOXO in male albino mice.

## Materials and methods

### Experimental animals

Fifty adult male albino mice (20–25 g) acquired from Theodor Bilharz Research Institute, Giza, Egypt, were employed. They were housed following the standards in the “Guide for the Care and Use of Laboratory Animals” prepared by the National Academy of Science, published by the National Institute of Health, and approved by the Animal House of Biochemistry, Chemistry Department, Faculty of Science, Damietta University, Egypt. Mice were conserved under controlled conditions (temperature of 23 ± 2 °C, appropriate humidity, and 12:12 h light/dark cycle) and fed with a standard mice pellet diet and water ad libitum.

### Experimental design

**Fig. 1:**
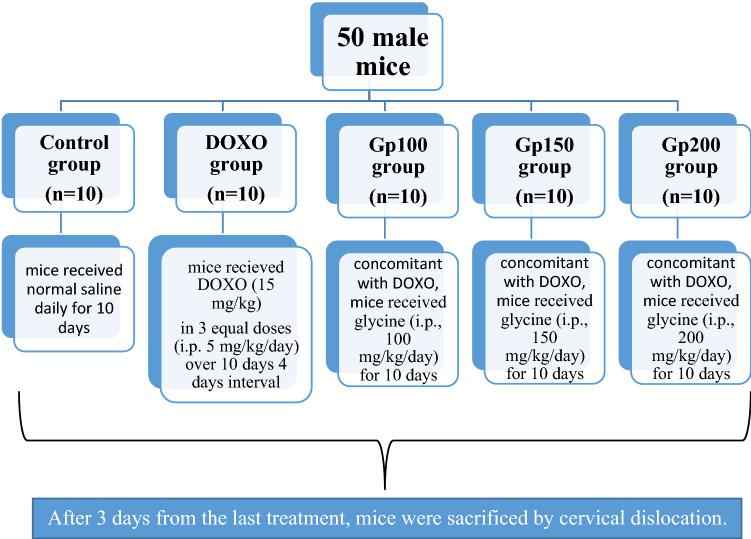
Schematic diagram for the experimental design

The effect of glycine treatment on cardiac toxicity induced by DOXO was studied by random dividing the fifty animals into five groups of ten animals each (Fig. 1) as follows:

1. Control group: mice received normal saline daily for ten days.
2. DOXO group [Cardiomyopathy (cardiac toxicity)]: mice received DOXO (Hikma Specialized Pharmaceuticals, Cairo, Egypt) (i.p., 5 mg/kg) in three equal injections over ten days (4 days intervals) for a cumulative dose of 15 mg/kg (Sahu et al. 2016).
3. Gp100 group: concomitant with DOXO, mice received glycine (El Nasr Pharmaceutical Chemicals, Cairo, Egypt) (i.p., 100 mg/kg/day) for ten days.
4. Gp150 group: concomitant with DOXO, mice received glycine (i.p., 150 mg/kg/day) for ten days.
5. Gp200 group: concomitant with DOXO, mice received glycine (i.p., 200 mg/kg/day) for ten days.

The food intake of groups was observed daily. Body weight was registered every five days over the experimental time to calculate the body weight difference (BWD). Three days later, after the last treatment, mice were fasted for eight h and sacrificed by cervical dislocation. Just before scarification, blood specimens (a mix of arterial and venous blood) were collected via cardiac puncture and left to clot. Clotted specimens were centrifuged for 15 min at 3000 rpm to separate sera. Finally, sera were stored at – 20 °C until used for estimations. The heart organ was quickly separated, washed with normal saline, blotted dry on filter papers, and weighed. Heart weight (HW)/body weight (BW) ratio was calculated: HW/BW *Ratio* = [HW (*g*)/BW (*g*)] × 100. Next, a part of the heart tissue was fixed in 10% buffered formalin for histological studies. Tissue homogenate (10%, W/V) was prepared in ice-cold PBS (pH 7.2) and centrifuged at 4000 rpm for 15 min at 4 °C. The resultant clear supernatant was used for biomarkers estimations in tissue.

### Biochemical estimations

Colorimetrically, the concentration of tissue malondialdehyde (MDA) as an indicator for lipid peroxides was estimated using the MDA kit purchased from BIODIAGNOSTIC, Giza, Egypt, based on the thiobarbituric acid method, GSH level was estimated by the GSH kit (BIODIAGNOSTIC, Giza, Egypt) depending on the breakdown of 5,5‾-dithiobis (2-nitrobenzoic acid) resulting in yellow color that measured at 405 nm, the activity of tissue superoxide dismutase (SOD) was assessed relying on the SOD’s ability to inhibit the phenazine methosulfate-mediated reduction of nitro blue tetrazolium dye as stated in the SOD kit (BIODIAGNOSTIC, Giza, Egypt), H_2_O_2_ degradation was the basis of measuring the activity of tissue catalase according to catalase kit (BIODIAGNOSTIC, Giza, Egypt), and tissue nitric oxide (NO) was estimated via applying the NO kit (BIODIAGNOSTIC, Giza, Egypt) procedures depending on the formation of a bright reddish purple azo-dye which can be measured at 540 nm.

Levels of TNF-α and interleukin-10 (IL-10) were measured in tissue based on the sandwich enzyme-linked immunosorbent assay (sandwich-ELISA) principle following the instructions of their commercial immunoassay kits (ELLABSCIENCE BIOTECHNOLOGY, USA).

Serum levels of uric acid, creatinine, urea, and creatine phosphokinase (CPK) were determined using kits obtained from BIOMED DIAGNOSTICS, Germany, according to the manufacturer’s procedure. In brief, creatinine and picric acid reacted in an alkaline medium, forming a yellow–orange color that was read at 492 nm using a colorimeter. By uricase, uric acid was oxidized to allantoin and H_2_O_2_. H_2_O_2_ reacted with 3,5-dichloro-2-hydroxy-benzene sulfonic acid and 4-amino antipyrine in the presence of peroxidase, forming a red dye that was read at 578 nm. By urease, urea was decomposed into ammonia and carbamic acid. Carbamic acid decomposed into NH_3_ and CO_2_. In an alkaline sodium hypochlorite solution, the released ammonium reacted with salicylate and nitroferricyanide, forming a green dye. The absorbance of the green color was read at 578 nm. CPK level was determined by following the rate of NADPH formation at 340 nm.

In addition, the rate of conversion of NADH/NAD^+^ at 340 nm was monitored to determine lactate dehydrogenase (LDH) activity using the LDH kit (Chema Diagnostica, Italy). Aspartate aminotransferase (AST) activity was measured using the kit from DIAMOND DIAGNOSTICS, Germany, depending on the measurement of the amount of oxalate formed through a definite time.

Calcium, phosphorus, sodium, and potassium kits (BIODIAGNOSTIC (diagnostic and research reagents), Giza, Egypt) instructions were followed for determination of their serum levels. Briefly, in an alkaline medium, calcium ions were let to react with methyl thymol blue forming a blue color, which was measured at 585 nm. In a reaction between phosphate and molybdic acid, the phosphomolybdate compound was formed that was reduced using SnCl_2_ to give a blue color that was measured at 640 nm. While potassium ions were let to react with sodium tetraphenyl boron to develop a colloidal solution, which was read at 420 nm. Regarding sodium level determination, sodium ions with excess uranyl acetate and magnesium acetate formed sodium magnesium uranyl acetate. The residual uranyl acetate was let to react with potassium ferrocyanide forming a colored complex, which was read at 545 nm.

### Histopathological assay

Fixed heart tissues were dehydrated by passing in ascending series of alcohol, cleared with xylene, and immersed in paraffin wax. Slices of the tissues of 5–6 μm thickness were prepared, stained with hematoxylin and eosin (H&E) dye, and examined under light microscopy. The pathologist assessing sections was uninformed of the mice’s treatment.

### Statistical analysis

Statistically, results were expressed as mean ± S.D., and the statistical analysis was performed using Instat software, version 3.10 (GraphPad, Inc., Sorrento Valley, San Diego, USA). For comparison between groups, the one-way analysis of variance (ANOVA) test was applied. *P* values of < 0.05, < 0.01, and < 0.001 were assumed significant, very significant, and extremely significant, respectively, for all analyses.

## Results

As shown in Table 1, the DOXO group displayed a significant weight reduction compared with the control mice group (17.53 ± 1.19 g for the DOXO group versus 27.57 ± 1.27 g for the control on day 10, *P* < 0.001) with a BWD of − 3.94 ± 0.9. Treated groups (Gp100, Gp150, and Gp200) displayed an approximate 30% increase in weight compared with DOXO alone with BWD of + 4.27 ± 0.72, + 5.37 ± 1.14, and + 5.1 ± 2.33, respectively. By comparing each glycine dose group with the other two glycine doses groups, all differences in mice weights on day 0, 5, or 10 were non-significant, except weight differences on day zero or day five between mice of Gp200 and Gp150 were significant (*P* < 0.001) compared to Gp100.

**Table 1:** Mice body weight changes (g) between mice groups throughout the experimental period starting from day 0 (first day of injection) up to day 10 (last day of injection) at different time intervals

Days	Day 0	Day 5	Day 10	BWD (Day10-Day0)
Control	24.42 ± 0.30	25.1 ± 0.19	27.57 ± 1.27	+ 3.15 ± 0.97 [**2.18 : 4.12**]
DOXO	21.47 ± 0.29***	19.77 ± 0.55***	17.53 ± 1.19***	− 3.94 ± 0.9 **[− 4.84 : − 3.04**]
Gp100	23 ± 0.94^***,!!!^	23.53 ± 0.73***^,!!!^	27.27 ± 1.66^a,!!!^	+ 4.27 ± 0.72 [**3.55 : 4.99**]
Gp150	21.35 ± 0.56^***,b,###^	21.82 ± 0.36***^,!!!,###^	26.72 ± 1.70^a,!!!,c^	+ 5.37 ± 1.14 [**4.23 : 6.51**]
Gp200	20.8 ± 0.4***^,b,###,d^	21.22 ± 0.39***^,!!!,###,d^	25.9 ± 2.73^a,!!!,c,d^	+ 5.1 ± 2.33 [**2.77 : 7.43**]

Results are expressed as mean± S.D, *n*=10 mice in each group. Bold values between square brackets represent BWD range. One-way ANOVA test was applied

*BWD* body weight difference

^a^*P* > 0.05 versus control group

***P < 0.001 versus control group

^b^*P* > 0.05 versus DOXO group

^!!!^ *P* < 0.001 versus DOXO group

^c^*P* > 0.05 versus Gp100 group

^###^*P* < 0.001 versus Gp100 group

^d^*P* > 0.05 versus Gp150 group. *P* > 0.05 is non-significant

Administration of doxorubicin in the DOXO group markedly lowered (*P* < 0.001) the HW by about 54% and HW/BW ratio by about 28% (0.051 g and 0.29 for the DOXO group versus 0.11 g and 0.4 for the control group). The HW raised significantly to approximately twofold in the glycine-treated groups as compared to the DOXO group (0.104 ± 0.0005, 0.104 ± 0.0009, and 0.109 ± 0.0008, respectively for Gp100, Gp150, and Gp200 versus 0.051 ± 0.0001 for the DOXO group). Also, HW/BW ratio depending on glycine dose was significantly elevated by about 31%, 34.5%, and 45% in Gp100, Gp150, and Gp200, respectively (from 0.29 in the DOXO group to 0.38, 0.39, and 0.42 for Gp100, Gp150, and Gp200, respectively). Comparing the HW and HW/BW ratio in each glycine dose group with the other two glycine doses groups, all differences were non-significant (Table 2).

**Table 2:**
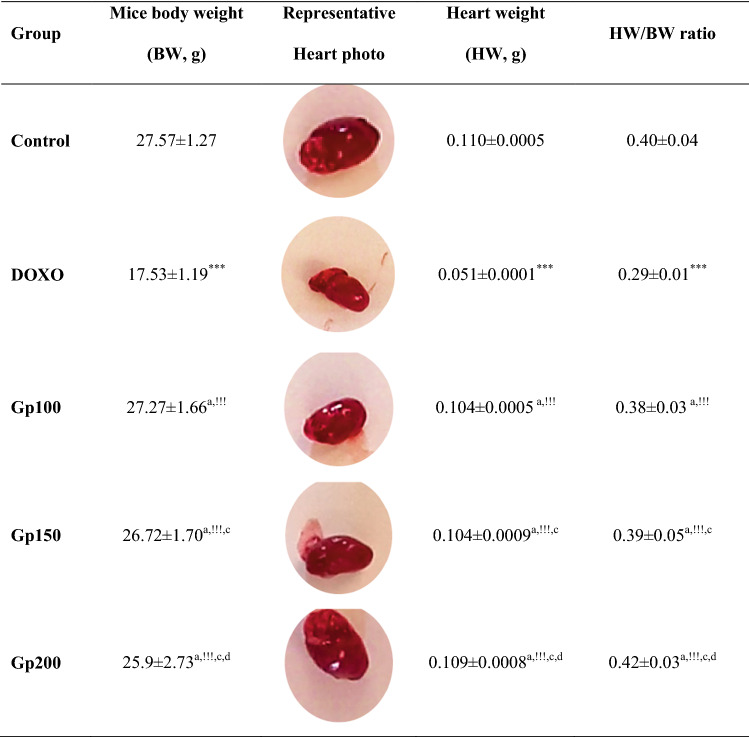
Differences in heart weight and heart weight/body weight ratio between mice groups on the day of scarification

Results are expressed as mean± S.D, *n*=10 mice in each group. One-way ANOVA test was applied

^a^*P* > 0.05 versus control group

^***^P < 0.001 versus control group

^b^*P* > 0.05 versus DOXO group

^!!!^*P* < 0.001 versus DOXO group

^c^*P* > 0.05 versus Gp100 group

^d^*P* > 0.05 versus Gp150 group. *P* > 0.05 is non-significant

**Fig. 2:**
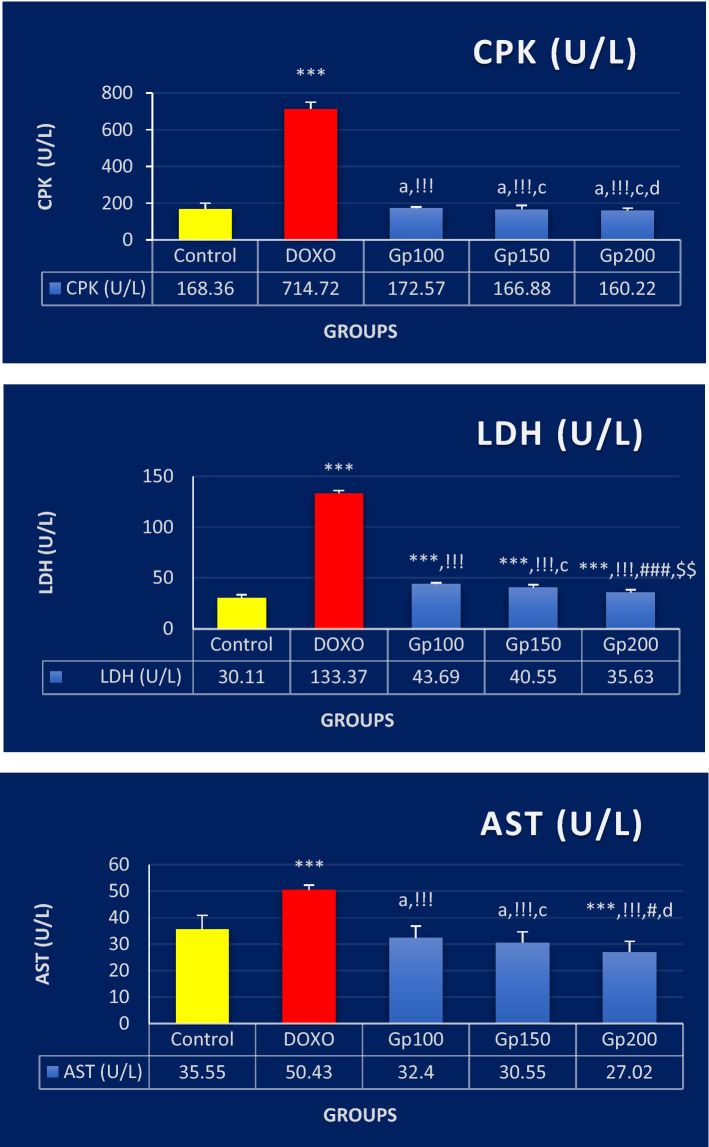
Bar graphs showing the changes in creatine phosphokinase (CPK), lactate dehydrogenase (LDH), and aspartate aminotransferase (AST) activities between different mice groups. Results are expressed as mean ± S.D, *n* = 10 mice in each group. One-way ANOVA test was applied. ^a,^ *^,^ ***P > 0.05, *P* < 0.05, *P* < 0.001, respectively versus control group. ^!!!^*P* < 0.001 versus DOXO group. ^c, #,^^###^*P* > 0.05, *P* < 0.05 and *P* < 0.001, respectively versus Gp100 group. ^d,^^$$^*P* > 0.05, *P* < 0.01, respectively versus Gp150 group. *P* > 0.05 is non-significant

In Fig. 2, the activities of LDH, CPK, and AST enzymes increased significantly (by about fourfold, fourfold, and onefold, respectively, *P* < 0.001) in the DOXO group compared to the control group. The treated groups (Gp100, Gp150, and Gp200) displayed, on the contrary, dose-dependent significant reductions in these activities. Respectively, Gp100 showed reductions by 67%, 76%, and 36%, Gp150 by 70%, 77%, and 39%, and Gp200 by 73%, 78%, and 46% when compared to the DOXO group (*P* < 0.001). There were non-significant differences found in the three enzymes when Gp100, Gp150, and Gp200 groups compared to each other except for AST in Gp200 when compared to that of Gp100 (Gp200 showed more reduction by 17%, *P* < 0.05) and for LDH in Gp200 when compared to Gp100 (Gp200 showed more reduction by 18%, *P* < 0.001) or to Gp150 (Gp200 showed more reduction by 12%, *P* < 0.01).

To explore the protection mechanisms of glycine on the heart, we determined the inflammation, oxidative stress, and antioxidant indices.

There were significant elevations (*P* < 0.001) in levels of TNF-α to about twofold (11.91 ± 0.59 ng/mL), IL-10 to about 11-fold (27.69 ± 0.57 ng/mL), MDA to about twofold (0.12 ± 0.013 nM/g ptn), and NO to about twofold (5.31 ± 0.42 mmol/L) in the DOXO group, compared to controls (5.38 ± 0.38, 2.63 ± 0.18, 0.065 ± 0.0043, and 2.39 ± 0.52, respectively). By treatment with glycine, in a dose-dependent manner, all these levels were decreased to the control/or to lower than the control range except for IL-10 levels were reduced but still higher than the normal control range in all the treated groups. TNF-α, IL-10, MDA, and NO levels were decreased by 45%, 82%, 42%, and 59%, respectively in Gp100, by 51%, 86%, 50%, and 71%, respectively in Gp150, and by 55%, 88%, 51%, and 74%, respectively in Gp200. Regarding comparing groups Gp100, Gp150, and Gp200 to each other, significantly IL-10 level was decreased by 24% in Gp150 and by 34% in Gp200 compared to Gp100 (*P* < 0.001), by 13% in Gp200 compared to Gp150 (*P* < 0.01), TNF-α level was declined by 11% in Gp150, and by 18% in Gp200 compared to Gp100 (*P* < 0.001), MDA level was decreased by 16% in Gp200 compared to Gp100 (*P* < 0.05), and NO level was reduced by 30% in Gp150, and by 38% in Gp200 compared to Gp100 (*P* < 0.01 and *P* < 0.001, respectively, Table 3).

**Table 3:** Differences in levels of IL-10, TNF-α, MDA, and NO between mice groups

Groups	IL-10 (ng/mL)	TNF-α (ng/mL)	MDA (nM/g ptn)	NO (mmol/L)
Control	2.63 ± 0.18	5.38 ± 0.38	0.065 ± 0.0043	2.39 ± 0.52
DOXO	27.69 ± 0.57***	11.91 ± 0.59***	0.12 ± 0.013***	5.31 ± 0.42***
Gp100	5.06 ± 0.09***^,!!!^	6.56 ± 0.29***^,!!!^	0.07 ± 0.003^a,!!!^	2.19 ± 0.57^a,!!!^
Gp150	3.84 ± 0.17^***,!!!,###^	5.85 ± 0.19*^,!!!,###^	0.0605 ± 0.0043^a,!!!,c^	1.53 ± 0.31***^,!!!,##^
Gp200	3.36 ± 0.36^***,!!!,###,$$^	5.40 ± 0.24^a,!!!,###,d^	0.0588 ± 0.005^a,!!!,#,d^	1.36 ± 0.19***^,!!!,###,d^

Results are expressed as mean± S.D, *n*=10 mice in each group. One-way ANOVA test was applied

^a^*P* > 0.05 versus control group

*P < 0.05 versus control group

***P < 0.001 versus control group

^!!!^*P* < 0.001 versus DOXO group

^c^*P* > 0.05 versus Gp100 group

^#^*P* < 0.05 versus Gp100 group

^##^*P* < 0.01 versus Gp100 group

^###^*P* < 0.001 versus Gp100 group

^d^*P* > 0.05 versus Gp150 group

^$$^*P* < 0.01 versus Gp150 group. *P* > 0.05 is non-significant

**Fig. 3:**
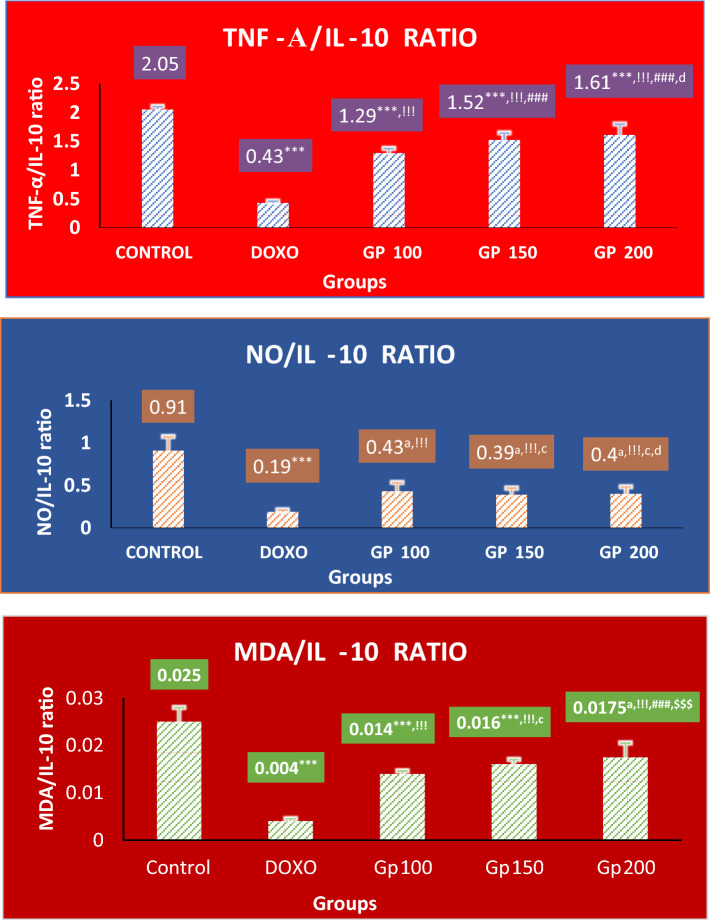
The ratio change of TNF-α/IL-10, MDA/IL-10, and NO/IL-10 between different mice groups. Ratio represents the value of mean level of M1 marker/mean level of M2 marker. Results are expressed as mean ± S.D, *n* = 10 mice in each group. One-way ANOVA test was applied. ^a,^ ***P > 0.05, *P* < 0.001, respectively versus control group. ^!!!^*P* < 0.001 versus DOXO group. ^c,^^###^*P* > 0.05, *P* < 0.001, respectively versus Gp100 group. ^d,^^$$$^*P* > 0.05, *P* < 0.001, respectively versus Gp150 group. *P* > 0.05 is non-significant. TNF-α: tumor necrosis factor-alpha, IL-10: Interleukin-10, MDA: malondialdehyde, NO: nitric oxide

Additionally, there were remarkable decreases in the ratios of M1/M2 macrophage polarization (TNF-α/IL-10, MDA/IL-10, and NO/IL-10 by 79%, 84%, and 79%, respectively) from 2.05 ± 0.05, 0.025 ± 0.003, and 0.91 ± 0.16, respectively in control to 0.43 ± 0.026, 0.004 ± 0.0005, and 0.19 ± 0.02, *P* < 0.001, respectively in the DOXO group, but they were re-elevated in the all treated groups. They were, respectively, re-elevated to 1.29 ± 0.08 (≈threefold), 0.014 ± 0.0006 (≈fourfold), and 0.43 ± 0.102 (≈twofold) in Gp100, to 1.52 ± 0.12 (≈fourfold), 0.016 ± 0.001 (≈fourfold), and 0.39 ± 0.08 (≈twofold) in Gp150, and 1.61 ± 0.18 (≈fourfold), 0.0175 ± 0.003 (≈fourfold), and 0.40 ± 0.081 (≈twofold) in Gp200. There were no significant differences noticed comparing NO/IL-10 changes in Gp100, Gp150, and Gp200 groups with each other. But for TNF-α/IL-10, only Gp150 and Gp200 showed significant increments by 18% and 25% compared to Gp100 (*P* < 0.001), while for MDA/IL-10, only Gp200 displayed a significant increase by 25% compared to Gp100 or by 9% compared to Gp150 (*P* < 0.001, Fig. 3).

Furthermore, the levels of GSH and uric acid, as well as the activities of SOD and catalase, exhibited significant drops by 79%, 23%, 36%, and 49% (17.37 µmol/L, 4.38 mg/dL, 23.003 % Inhibition, and 282.75 U/g ptn, respectively, *P* < 0.001) in the DOXO group compared to the control group (84.39 µmol/L, 5.71 mg/dL, 35.79 % Inhibition, and 551.79 U/g ptn, respectively). Glycine treatment caused elevations in antioxidants in a concentration-dependent manner. By glycine treatment, the SOD level was significantly increased to a level higher than that in both the DOXO and control groups. These increments were by 74%, 76%, and 84% in Gp100, Gp150, and Gp200, respectively, compared to the DOXO group, and by 12%, 13%, and 19%, respectively, compared to the control group. GSH level was also increased to about threefold compared to the DOXO group (*P* < 0.001), but to a level still lower than the control (47.42, 50.92, and 52.2 for Gp100, Gp150, and Gp200 groups, respectively, versus 84.39 µmol/L for the control group). Catalase and uric acid levels as well showed a dose-dependent increase within the glycine-treated groups, but their ranges were still lower than those in the control and DOXO groups. Changes in the levels of GSH, uric acid, SOD, or catalase between the Gp100, Gp150, and Gp200 groups were statistically non-significant when each group was compared to the other two (Table 4).

**Table 4:** Differences of the levels of SOD, catalase, GSH, and uric acid of different mice groups

Groups	SOD (% Inhibition)	Catalase (U/g ptn)	GSH (µmol/L)	Uric acid (mg/dL)
Control	35.79 ± 2.07	551.79 ± 90.26	84.39 ± 8.12	5.71 ± 0.62
DOXO	23.003 ± 1.12***	282.75 ± 46.48***	17.37 ± 1.66***	4.38 ± 0.67***
Gp100	40.11 ± 3.08*^,!!!^	183.65 ± 0.82***^,!!!^	47.42 ± 2.58***^,!!!^	3.371 ± 0.63***^,!!^
Gp150	40.46 ± 1.78**^,!!!,c^	184.72 ± 0.69***^,!!!,c^	50.92 ± 3.44***^,!!!,c^	3.723 ± 0.34***^,b,c^
Gp200	42.44 ± 4.17***^,!!!,c,d^	198.75 ± 8.62***^,!!!,c,d^	52.2 ± 8.092***^,!!!,c,d^	3.79 ± 0.23***^,b,c,d^

Results are expressed as mean± S.D, *n*=10 mice in each group. One-way ANOVA test was applied

*P < 0.05 versus control group

**  *P* < 0.01 versus control group

***P < 0.001 versus control group

^b^*P* > 0.05 versus DOXO group

^!!^*P* < 0.01 versus DOXO group

^!!!^*P* < 0.001 versus DOXO group

^c^*P* > 0.05 versus Gp100 group

^d^*P* > 0.05 versus Gp150 group. *P* > 0.05 is non-significant

**Fig. 4:**
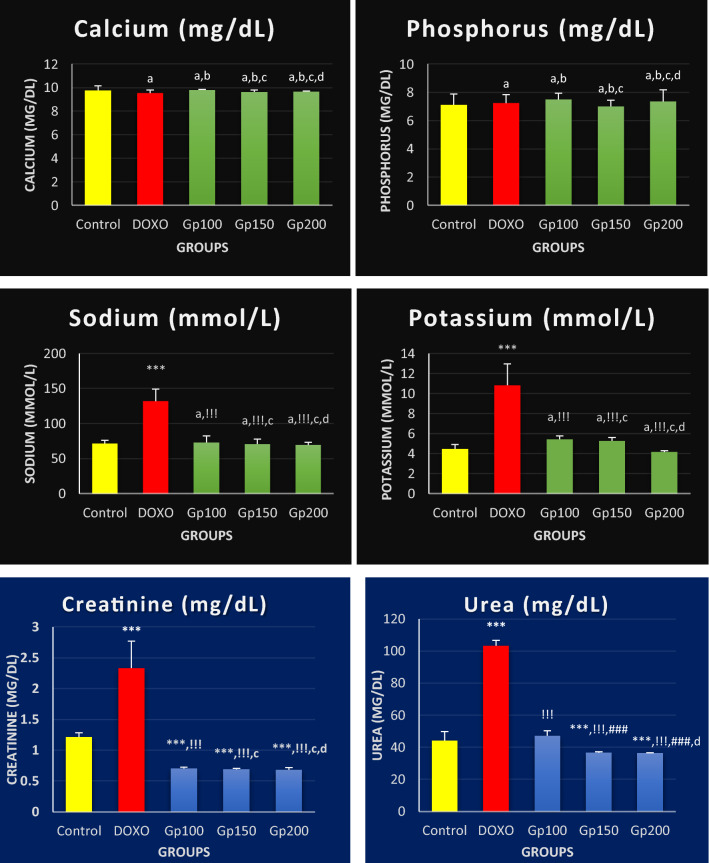
Bar graphs showing calcium, phosphorus, sodium, potassium, creatinine, and urea concentrations in sera of different mice groups. Results are expressed as mean ± S.D, *n* = 10 mice in each group. One-way ANOVA test was applied. ^a,^ ***P > 0.05, *P* < 0.001, respectively versus control group. ^b,^^!!!^P > 0.05, P < 0.001, respectively versus DOXO group. ^c,^^###^*P* > 0.05, *P* < 0.001, respectively versus Gp100 group. ^d^*P* > 0.05 versus Gp150 group. P > 0.05 is non-significant

Figure 4 illustrates that non-significant differences were found in Ca and P concentrations in all the studied groups compared to the control group. Concerning Na, K, urea, and creatinine concentrations, they showed significant elevations to approximately twofold (131.89 mmol/L, 10.83 mmol/L, 103.32 mg/dL, and 2.33 mg/dL, respectively, *P* < 0.001) in the DOXO group, which were significantly reduced by glycine treatment, depending on glycine concentration. Na, K, urea, and creatinine, respectively, showed reduced levels by about 45%, 50%, 54%, 70% in Gp100; 46%, 51%, 65%, and 70% in Gp150; and 47%, 61%, 65%, and 71% in Gp200. In Fig. 4, only the urea levels in groups Gp150 and Gp200 were significantly reduced by 22% and 23%, respectively, compared to its level in Gp100 (*P* < 0.001); all comparisons between each glycine dose group, Gp100, Gp150, or Gp200, and the other two groups in the levels of Ca, P, Na, K, and creatinine were statistically non-significant.

**Fig. 5:**
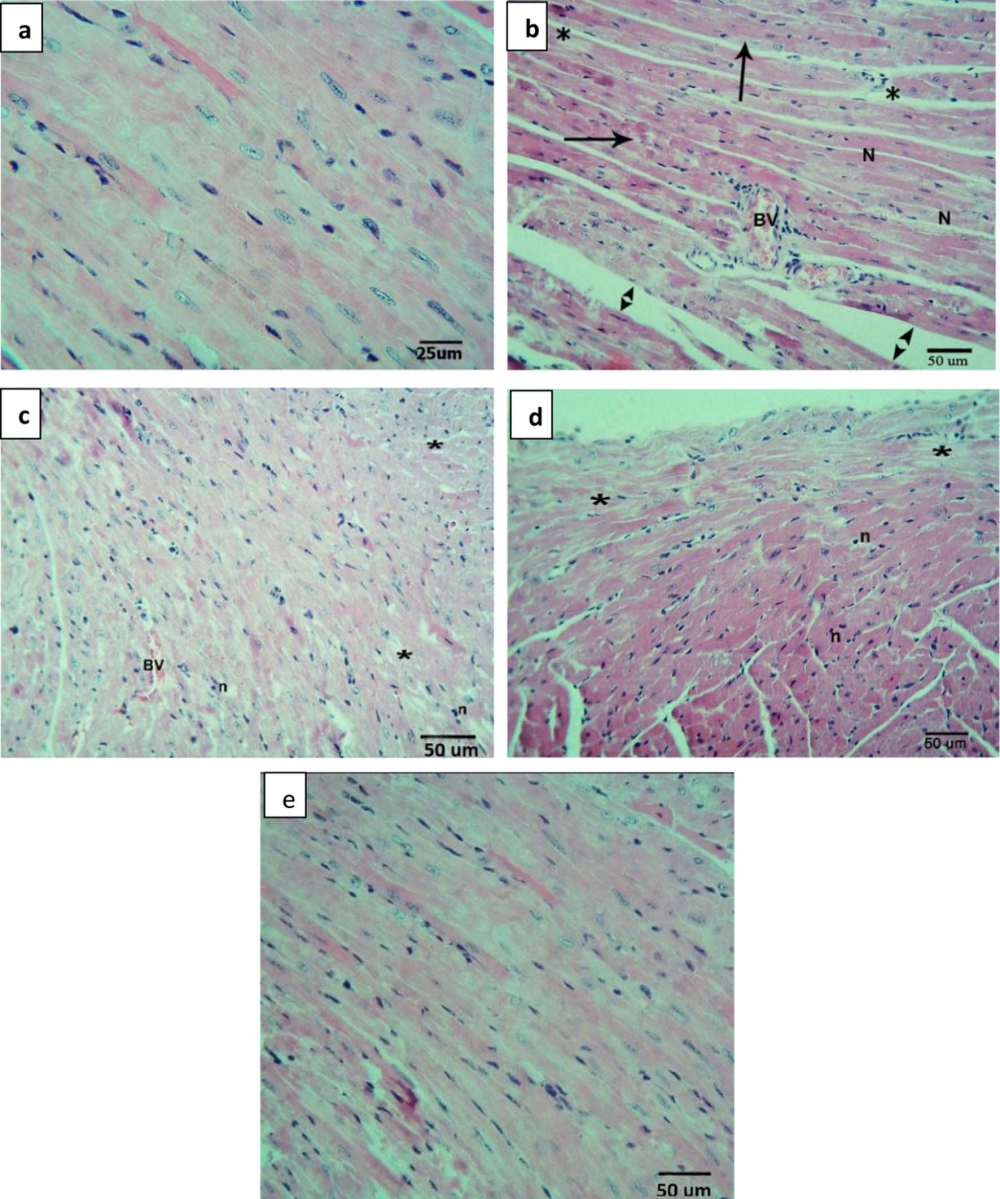
Heart histopathology of all studied groups (Hematoxylin and eosin). Control group **(a)** Section showing the myocardial tissue structure was clear, show a normal myofibrillar structure, cell arrangements were orderly and exhibited clear nuclei, the cell membrane was intact and no pathological change was observed. The cardiac myofibers running parallel to the apical–basal axis. DOXO group (**b)** Section showing severe affection of the myocardium with dispersed myofibrils with patchy areas of pale stained fibers with loss of the normal alignment separated by blood vessels (BV). The individual fibers were multinucleated (N). There were obvious myocardial cell swelling, widened muscle space of cardiac myocytes and areas of intervening edema, congestion, necrosis (*), inflammatory cell infiltration, and showed myocardial cell damage. Wide spaces are marked by the distance between arrowheads and vacuolated cytoplasm (arrow). Gp100 (**c)** Section showing apparent mild cell stress in the form of dispersed myofibrils (stars) surrounding congested blood vessel (BV). Some fibers show pyknotic nuclei (apoptosis) (n). Gp150 **(d)** Section showing normal cardiac myofibers running parallel to the apical–basal in the form of few dispersed myofibrils (stars). Some fibers show pyknotic nuclei (apoptosis) (n). Gp200 **(e)** Section showing apparent normal cardiac myofibers running parallel to the apical–basal with a picture more close to control group

Histopathologically, heart tissue in the control group showed normal histology (Fig. 5a). Heart tissue in the DOXO group showed myocardial cell damage. Cardiac toxicity manifested by congestion of myocardial cells associated with necrosis and inflammatory cell infiltration (Fig. 5b). Heart tissues of treated groups showed gradual normalization of damage of heart tissues depending on glycine dose. Remarkably, the heart tissues of Gp100 (Fig. 5c) showed apparent mild to moderate affection of cardiac myofibers, Gp150 (Fig. 5d), and Gp200 (Fig. 5e) showed the best normalization.

## Discussion

In the current study, DOXO injection seemed connected with substantial declined body weight gain, collapsed heart, and low HW/BW ratio as outcomes of the cardiac damage linked with DOXO-induced toxicity ascertained by the congestion of the heart, necrosis, severe inflammation, and inflammatory cell infiltration seen in the histopathology of heart sections. These marked drops might be due to increased catabolism because of declined food intake attributed to the noticed loss of appetite and fatigue. This is in agreement with Abdelatty et al. (2021) and Ikewuchi et al. (2021). Increases in serum urea and creatinine, as well as decreased total protein (data not shown), are supportive of the suggested increased catabolism. This is in harmony with Carlotti et al. (2008). On the other side, all treated groups with glycine gradually gained body weight and restored their near normal/normal heart weight, body weight, and HW/BW ratio at the end of the experiment based on the utilized dose. These findings reflect that glycine was able to improve appetite and encourage anabolism. The glycine dose of 200 mg/kg exerted the most favorable outcome.

CPK and LDH leak mainly from damaged cardiac tissue. AST chiefly leaks from damaged hepatic tissues but also leeks from other damaged tissues, including cardiac tissues (Djakpo et al. 2020). DOXO-induced heart toxicity, in the current study, resulted in a distinctive elevation in the activities of the three enzymes relative to the control mice, which is in harmony with Al‑Harthi et al. 2014 and Cui et al. (2021), indicating damaging of tissues. CPK and LDH elevations, aside from the increase in AST level, were also observed in Ehrlich-induced oxidative tissue damage (Saad et al. 2022). Moreover, Ewid et al. (2020) and Yokoyama et al. (2016) indicated a close association of AST with cardiovascular disease, and different mechanisms were proposed for the elevated level, for example, increased plasma inflammatory mediators and oxidative stress. In the same line, we attribute these enzymatic elevations to the oxidative stress-induced increased permeability of the damaged-tissue membranes, chiefly heart and liver tissues.

According to Kong et al. (2022), DOXO promotes oxygen absorption increment and creates numerous reactive oxygen and nitrogen species, leading to oxidative stress. DOXO-induced oxidative stress is counted as the leading reason for cardiotoxicity. The myocardium produces low levels of antioxidants, such as SOD, catalase, and GSH, making it exceedingly exposed to DOXO-induced cardiac damage. DOXO-induced-reactive species decline the production of antioxidants and inhibit their activities in a time and dose-based mode. Uric acid is one of the primary endogenous antioxidants in the body’s defense system; it comprises up to 60% of the plasma antioxidant capacity. Thus, uric acid is a reactive species scavenger, and thereby it is considered a cellular protector against oxidative stress; it interacts promptly with different reactive species, e.g., singlet oxygen, NO, and H_2_O_2_ giving neutralized intermediates (Yu and Cheng 2020). Some researchers reported uric acid cardio-protective effects. For example, in chicken embryo cardiomyocytes, according to Sun et al. (2017), at its physiological level, uric acid was able to decline oxidative stress-induced MDA and protein carbonyl contents, reinforce SOD activity, and block the production of reactive species. In perfused guinea pig myocardium, at its physiological level, uric acid was able to ameliorate heart functional responses and stability that were impaired by oxidants (Becker et al. 1989).

In many tissue toxicity models, toxicity-induced oxidative stress has been emphasized (El-Shahat et al. 2021; Habib et al. 2020; Saad et al. 2019). In the present work, toxicity-induced oxidative stress was also evident in our cardiac toxicity model. MDA and NO were elevated in association with reductions in antioxidants (SOD, catalase, GSH, and uric acid) in mice with untreated cardiotoxicity, indicating an oxidative stress state. Oxidative stress results in the cell membrane poring and tissue damage, causing tissue enzyme elevations in serum. In agreement with Sheela and Shyamala (2000), the damage to the myocardial cell membrane increases its permeability with subsequent leakage of heart tissue enzymes into the bloodstream. This deterioration was symptomized obviously in DOXO group heart tissue sections, as mentioned above. There was perspicuous heart cell swelling, which translated into elevated levels of sodium and potassium minerals, widened muscle space of cardiac myocytes and areas of intervening edema, congestion, and necrosis where oxygenation is poor, as well as inflammatory cell infiltration, which remarkably translated into elevated levels of pro-inflammatory parameters (TNF-α and NO), this complies with Szabo et al. (2021). In our study, glycine treatment showed improvements toward normalization of these levels suggesting that glycine is a powerful heart tissue protector against heart injury induced by DOXO. These are perfectly compatible with histopathology findings as the architecture of the heart tissue shown with treatment with a glycine dose of 200 mg was close to normal. Lu et al. (2017) stated glycine exhibits a cardio-protective impact by decreasing serum cardiac markers.

Inflammation is joined instantly with cytokines secretions, such as TNF-α, secreted by macrophages, monocytes, T lymphocytes, and mast cells (Grabarek et al. 2017). Aside from its well-recognized pro-inflammatory function (Toson et al. 2022), TNF-α has intricate influences on the evolution, differentiation, and death of the immune cells (Saad et al. 2022). The TNF-α pivotal impact is connected with its binding to receptors TNFR1 and TNFR2. Most reports demonstrate a critical role for TNF-α in the two leading causes of death, cancers (Aboseada et al. 2021) and cardiovascular diseases (Saad et al. 2020), despite medical progress and public awareness of protection (Grabarek et al. 2017). Reactive species and other inflammatory stimuli invigorate particular receptors and begin intracellular signaling that will produce pain and inflammation. In the state of inflammation, immune cells secrete TNF-α with other mediators that provoke nociceptors resulting in pain inducement (Ferraz et al. 2020). Anti-inflammatory IL-10 is secreted by macrophages in varied immune conditions and is pivotal in limiting immune-mediated pathology (Sanin et al. 2015). Inflammatory biomarker NO (Manisundar et al. 2014) is an inflammatory mediator. Macrophages and other inflammatory cells promote its synthesis and release. Its content may announce the severity and status of the implied disease process (Bhatia et al. 2003). In harmony with our findings, Shaker et al. (2018) found significant rises in TNF-α and MDA in the myocardium after DOXO injection. They also mentioned that numerous earlier documents emphasized that DOXO causes the flow of inflammatory cytokines, including TNF-α, and they added the excessive increase of pro-inflammatory cytokines was proposed, by other researchers, as a main pathological reason behind DOXO-induced cardiac damage. Clinically, Alves et al. (2022) also established that breast cancer patients who suffered from DOXO therapy-induced cardiotoxicity showed elevations in plasma IL-10 concentrations. In harmony with Saad et al. (2019), disturbance in IL-10 levels is possible through the cytotoxic influence of the drug on leukocytes. Parallel to our findings, high sera levels of IL-10 and TNF-alpha were associated with admitted patients with myocarditis and those with acute myocardial infarction (Nishii et al. 2004).

Macrophages participate a pivotal role in monitoring immunity and immunomodulation; this can be monitored by evaluating M1 and M2 markers. M1/M2 symbolizes the two leading and antagonistic activities of macrophages. The activity of M1 prohibits cell proliferation and results in tissue harm, whereas M2 activity encourages cell proliferation and tissue remedy, according to Mills (2012). M1 macrophages produce pro-inflammatory cytokines (such as IL-1β, TNF-α, and IL-6), phagocytize microbes (e.g., bacteria and viruses), and begin an immune response. M1 macrophages produce NO or reactive oxygen intermediates to defend against microbes (Fukui et al. 2018). Thereby, TNF-α, NO, and MDA can be considered M1 markers. Conversely, M2 macrophages are immune cells that polarized in response to Th2-produced cytokines (e.g., interleukins 4, 5, 9, 10, 13, and 25) for mediation of the activation and sustain of the humoral, or antibody-mediated, the immune response against parasites, microbes, allergens, and toxins (Fukui et al. 2018). Thereby, IL-10 can be considered an M2 marker. IL-10 is a potent anti-inflammatory cytokine; it strongly inhibits the production of pro-inflammatory cytokines, so it can prohibit pathogen clearance and recover immunopathology (Couper et al. 2008).

Since M1 markers, including NO, TNF-α, and MDA, mean levels were increased by DOXO injection in the current work but decreased by glycine co-treatment, one can suggest that M1 polarization was activated by DOXO and suppressed by glycine. Contrary to our expectations, by looking at the M1/M2 ratio values, decreases were associated with DOXO, and increases were associated with glycine groups indicating the dominance of M2 polarization in the case of DOXO intoxication and the reverse with glycine co-treatment. The polarization change induced by glycine was more pronounced at the highest dose (200 mg). In this regard, one can assume that during uncontrollable cardiotoxicity stimulated by DOXO, most of the macrophages have switched to the M2 phenotype, the more anti-inflammatory phenotype, which assists in tissue remodeling (Akata and van Eeden 2020), to cope with the inflammation stress burden ascertained by marked increases in NO, TNF-α, and MDA in a trial from the host body to protect itself against the interactions initiated via DOXO intoxication, which is likely to show immunosuppression potentials. The dramatic elevation in IL-10 in the DOXO group is evidence. IL-10 is a cytokine with potent anti-inflammatory properties that plays a central role in limiting the host's immune response to pathogens in a trial to face the elevated pro-inflammatory markers, prevent damage to the host, and maintain normal tissue homeostasis. In contrast, glycine co-treatment showed the power to neutralize DOXO toxicity as it kept pro-inflammatory (TNF-α, MDA, and NO) levels within their normal limits and, at the same time, reduced anti-inflammatory IL-10 to a little bit higher level than healthy control to assure damage prevention and maintenance of normal tissue homeostasis. Therefore, in our model, glycine co-administration succeeded in retrieving the polarization dominance back to its healthy control normal state (classic M1 promotion), which shows immune reactivation potential that may help the host body prevent/face the worsened symptoms of DOXO-induced cardiotoxicity classically if present.

In heart failure, Na and K are the widely disturbed electrolytes. Na is required for the maintenance of typical blood volume and pressure. Association is established between hypernatremia and developing stroke and cardiovascular diseases (Patel and Joseph 2020). Wang et al. (2019) reported high serum Na levels in the DOXO-intoxicated group compared to the normal and attributed this elevation to reduced Na renal reabsorption due to tubular disorders. Increased serum Na levels were also seen in rats having cardiac damage (Lossnitzer and Bajusz 1974). Concerning K levels, hyperkalemia is frequent in patients with heart failure due to different reasons, like neurohormonal alterations implicated in the illness and kidney dysfunction, and is associated with a higher risk of cardiovascular complications (Rakisheva et al. 2020). Hyperkalemia is also markedly related to poor prognosis in those patients and may influence survival (Toledo et al. 2021). Other studies, like Jung et al. (2022), reported increased Ca and P levels in heart failure. In the current study, DOXO intoxication did not alter Ca and P serum levels but showed marked increases in serum Na and K levels that might be due to DOXO-induced renal complications or/and neurohormonal disturbances behind cardiotoxicity pathogenesis. In our opinion, DOXO-induced renal complications are expected, as DOXO, in a dose of 15 mg/kg or18 mg/kg, was previously used by Habib et al. (2020) and Afsar et al. (2020) to induce chronic renal failure or kidney dysfunction disease in their rat models. Elevated urea and creatinine levels in our current study are further evidence of DOXO-induced kidney dysfunction. Glycine co-treatment restored Na & K with urea and creatinine toward their normal ranges indicating amelioration of DOXO-induced deterioration. This amelioration was supported further by histopathology as the heart tissue of the Gp200 group revealed normal cardiac myofibers with a picture close to the control group.

A limitation of our study is that we could not perform Echocardiography to evaluate cardiac function.

In conclusion, DOXO-induced heart toxicity displayed a marked lowering in HW/BW ratio, significant elevations in the activities of CPK, AST, and LDH enzymes, significant elevations in Na, K, TNF-α, IL-10, MDA, and NO levels, and significant drops in levels of GSH and SOD. Glycine treatment reversed all these levels toward normal in a dose-dependent manner. Furthermore, histopathology findings were supportive of the biochemical findings. In addition, our findings indicate the dominance of M1 over M2 in control and glycine-treated groups but the dominance of M2 in the DOXO group. Upon all findings, the present study demonstrated that glycine protected mice’s myocardium against DOXO-induced cardiotoxicity by modulating oxidative stress, inflammation, and immunity. It seems that the anti-oxidative criteria of glycine are behind its exerted positive actions. Finally, we introduce glycine as a potentially good option against DOXO-induced myocardial damage.

## Data Availability

Datasets generated in the current study are available from the corresponding author on reasonable request.

## References

[CR1] Abdelatty A, Ahmed MS, Abdel-Kareem MA (2021). Acute and delayed doxorubicin-induced myocardiotoxicity associated with elevation of cardiac biomarkers, depletion of cellular antioxidant enzymes, and several histopathological and ultrastructural changes. Life (basel).

[CR2] Aboseada HA, Hassanien MM, El-Sayed IH, Saad EA (2021). Schiff base 4-ethyl-1-(pyridin-2-yl) thiosemicarbazide up-regulates the antioxidant status and inhibits the progression of Ehrlich solid tumor in mice. Biochem Biophys Res Commun.

[CR3] Afsar T, Razak S, Almajwal A, Al-Disi D (2020). Doxorubicin-induced alterations in kidney functioning, oxidative stress, DNA damage, and renal tissue morphology; Improvement by *Acacia hydaspica* tannin-rich ethyl acetate fraction. Saudi J Biol Sci.

[CR4] Akata K, van Eeden SF (2020). Lung macrophage functional properties in chronic obstructive pulmonary disease. Int J Mol Sci.

[CR5] Al-Harthi SE, Alarabi OM, Ramadan WS (2014). Amelioration of doxorubicin-induced cardiotoxicity by resveratrol. Mol Med Rep.

[CR6] Alves MT, Simões R, Pestana RMC (2022). Interleukin-10 levels are associated with doxorubicin-related cardiotoxicity in breast cancer patients in a one-year follow-up study. Immunol Invest.

[CR7] Becker BF, Reinholz N, Ozcelik T, Leipert B, Gerlach E (1989). Uric acid as radical scavenger and antioxidant in the heart. Pflugers Arch.

[CR8] Bhatia S, Shukla R, Madhu SV, Gambhir JK, Prabhu KM (2003). Antioxidant status, lipid peroxidation and nitric oxide end products in patients of type 2 diabetes mellitus with nephropathy. Clin Biochem.

[CR9] Cardinale D, Iacopo F, Cipolla CM (2020). Cardiotoxicity of anthracyclines. Front Cardiovasc Med.

[CR10] Carlotti AP, Bohn D, Matsuno AK, Pasti DM, Gowrishankar M, Halperin ML (2008). Indicators of lean body mass catabolism: emphasis on the creatinine excretion rate. QJM.

[CR11] Chatterjee K, Zhang J, Honbo N, Karliner JS (2010). Doxorubicin cardiomyopathy. Cardiology.

[CR12] Couper KN, Blount DG, Riley EM (2008). IL-10: the master regulator of immunity to infection. J Immunol.

[CR13] Cui J, Shi Y, Xu X, Zhao F, Zhang J, Wei B (2021). Identifying the cardioprotective mechanism of Danyu Tongmai granules against myocardial infarction by targeted metabolomics combined with network pharmacology. Phytomedicine.

[CR14] Ding Y, Svingen GF, Pedersen ER (2015). Plasma glycine and risk of acute myocardial infarction in patients with suspected stable angina pectoris. J Am Heart Assoc.

[CR15] Djakpo DK, Wang ZQ, Shrestha M (2020). The significance of transaminase ratio (AST/ALT) in acute myocardial infarction. Arch Med Sci Atheroscler Dis.

[CR16] El Hafidi M, Pérez I, Baños G (2006). Is glycine effective against elevated blood pressure?. Curr Opin Clin Nutr Metab Care.

[CR17] El-Shahat RA, El-Demerdash RS, El Sherbini ES, Saad EA (2021). HCl-induced acute lung injury: a study of the curative role of mesenchymal stem/stromal cells and cobalt protoporphyrin. J Genet Eng Biotechnol.

[CR18] Ewid M, Sherif H, Allihimy AS (2020). AST/ALT ratio predicts the functional severity of chronic heart failure with reduced left ventricular ejection fraction. BMC Res Notes.

[CR19] Ferraz CR, Carvalho TT, Manchope MF (2020). Therapeutic potential of flavonoids in pain and inflammation: Mechanisms of action, pre-clinical and clinical data, and pharmaceutical development. Molecules.

[CR20] Fukui S, Iwamoto N, Takatani A (2018). M1 and M2 monocytes in rheumatoid arthritis: a contribution of imbalance of M1/M2 monocytes to osteoclastogenesis. Front Immunol.

[CR21] Grabarek B, Bednarczyk M, Mazurek U (2017). The characterization of tumor necrosis factor alpha (TNF-α), its role in cancerogenesis and cardiovascular system diseases and possibilities of using this cytokine as a molecular marker. Acta Universitatis Lodziensis.

[CR22] Habib SA, Saad EA, Al-Mutairi FM, Alalawy AI, Sayed MH, El-Sadda RR (2020). Up-regulation of antioxidant status in chronic renal failure rats treated with mesenchymal stem cells and hematopoietic stem cells. Pak J Biol Sci.

[CR23] Ikewuchi CC, Ikewuchi JC, Ifeanacho MO (2021). Protective effect of aqueous leaf extracts of *Chromolaena odorata* and *Tridax procumbens* on doxorubicin-induced hepatotoxicity in Wistar rats. Porto Biomed J.

[CR24] Jarvis S (2019). Cardiomyopathies 1: classification, pathophysiology and symptoms. Nurs times [online].

[CR25] Jarvis S, Saman S (2018). Cardiac system 1: anatomy and physiology. Nurs times [online].

[CR26] Jung DH, Park B, Lee YJ (2022). Longitudinal effects of serum calcium and phosphate levels and their ratio on incident ischemic heart disease among Korean adults. Biomolecules.

[CR27] Kalyanaraman B (2020). Teaching the basics of the mechanism of doxorubicin-induced cardiotoxicity: have we been barking up the wrong tree?. Redox Biol.

[CR28] Kong CY, Guo Z, Song P (2022). Underlying the mechanisms of doxorubicin-induced acute cardiotoxicity: oxidative stress and cell death. Int J Biol Sci.

[CR29] Li X, Zhu X, Chen Q (2015). Protective effect of glycine in cardiovascular diseases. Adv Biochem Biophy.

[CR30] Lossnitzer K, Bajusz E (1974). Water and electrolyte alterations during the life course of the BIO 14.6 Syrian golden hamster. A disease model of a hereditary cardiomyopathy. J Mol Cell Cardiol.

[CR31] Lu Y, Zhu X, Li J (2017). Glycine prevents pressure overload induced cardiac hypertrophy mediated by glycine receptor. Biochem Pharmacol.

[CR32] Manisundar N, Julius A, Amudhan A, Hemalatha VT, Manigandan T (2014). Nitric oxide as an inflammatory biomarker in oral and systemic diseases-a systematic review Middle-East. J Sci Res.

[CR33] Metra M, Teerlink JR (2017). Heart failure. Lancet.

[CR34] Mills CD (2012). M1 and M2 macrophages: oracles of health and disease. Crit Rev Immunol.

[CR35] Nishii M, Inomata T, Takehana H (2004). Serum levels of interleukin-10 on admission as a prognostic predictor of human fulminant myocarditis. J Am Coll Cardiol.

[CR36] Patel Y, Joseph J (2020). Sodium intake and heart failure. Int J Mol Sci.

[CR37] Pérez-Torres I, Zuniga-Munoz AM, Guarner-Lans V (2017). Beneficial effects of the amino acid glycine. Mini Rev Med Chem.

[CR38] Rakisheva A, Marketou M, Klimenko A, Troyanova-Shchutskaia T, Vardas P (2020). Hyperkalemia in heart failure: foe or friend?. Clin Cardiol.

[CR39] Roberts WC, Haque S, Hall SA (2018). Total 12-Lead QRS voltage in patients having orthotopic heart transplantation for heart failure caused by adriamycin-induced cardiomyopathy. Cardiol.

[CR40] Ruiz-Ramírez A, Ortiz-Balderas E, Cardozo-Saldaña G, Diaz-Diaz E, El-Hafidi M (2014). Glycine restores glutathione and protects against oxidative stress in vascular tissue from sucrose-fed rats. Clin Sci (lond).

[CR41] Saad EA, El-Demerdash RS, Abd EI-Fattah EM (2019). Mesenchymal stem cells are more effective than captopril in reverting cisplatin-induced nephropathy. Biocell.

[CR42] Saad EA, Kiwan HA, Hassanien MM, Al-Adl HE (2020). Synthesis, characterization, and antitumor activity of a new iron-rifampicin complex: a novel prospective antitumor drug. J Drug Deliv Sci Technol.

[CR43] Saad EA, Zahran F, El-Ablack FZ, Eleneen AMA (2022). A newly synthesized derivative and a natural parent molecule: Which would be more beneficial as a future antitumor candidate? Docking and in vivo study. Appl Biochem Biotechnol.

[CR44] Sahu BD, Kumar JM, Kuncha M, Borkar RM, Srinivas R, Sistla R (2016). Baicalein alleviates doxorubicin-induced cardiotoxicity via suppression of myocardial oxidative stress and apoptosis in mice. Life Sci.

[CR45] Sanin DE, Prendergast CT, Mountford AP (2015). IL-10 production in macrophages is regulated by a TLR-driven CREB-mediated mechanism that is linked to genes involved in cell metabolism. J Immunol.

[CR46] Shaker RA, Abboud SH, Assad HC, Hadi N (2018). Enoxaparin attenuates doxorubicin induced cardiotoxicity in rats via interfering with oxidative stress, inflammation and apoptosis. BMC Pharmacol Toxicol.

[CR47] Sheela SC, Shyamala DCS (2000). Protective effect of Abana®, a poly-herbal formulation, on isoproterenol-induced myocardial infarction in rats. Indian J Pharmacol.

[CR48] Sun X, Jiao H, Zhao J, Wang X, Lin H (2017). Unexpected effect of urate on hydrogen peroxide-induced oxidative damage in embryonic chicken cardiac cells. Free Radic Res.

[CR49] Szabo TM, Frigy A, Nagy EE (2021). Targeting mediators of inflammation in heart failure: a short synthesis of experimental and clinical results. Int J Mol Sci.

[CR50] Toledo CC, Vellosa Schwartzmann P, Miguel Silva L (2021). Serum potassium levels provide prognostic information in symptomatic heart failure beyond traditional clinical variables. ESC Heart Fail.

[CR51] Toson EA, Saad EA, Omar HAE (2022). Occupational exposure to gasoline in gasoline station male attendants promotes M1 polarization in macrophages. Environ Sci Pollut Res Int.

[CR52] Wang Y, Chao X, Ahmad FUD, Shi H, Mehboob H, Hassan W (2019). Phoenix dactylifera protects against doxorubicin-induced cardiotoxicity and nephrotoxicity. Cardiol Res Pract.

[CR53] Word Health Organization (WHO) (2021) Cardiovascular diseases (CVDs) https://www.who.int/news-room/fact-sheets/detail/cardiovascular-diseases-(cvds). Accessed 11 June 2021

[CR54] Xie S, Zhou W, Tian L, Niu J, Liu Y (2016). Effect of N-acetyl cysteine and glycine supplementation on growth performance, glutathione synthesis, anti-oxidative and immune ability of Nile tilapia, *Oreochromis niloticus*. Fish Shellfish Immunol.

[CR55] Yokoyama M, Watanabe T, Otaki Y (2016). Association of the aspartate aminotransferase to alanine aminotransferase ratio with BNP level and cardiovascular mortality in the general population: the yamagata study 10-year follow-up. Dis Markers.

[CR56] Yu W, Cheng JD (2020). Uric acid and cardiovascular disease: an update from molecular mechanism to clinical perspective. Front Pharmacol.

[CR57] Zamorano JL, Lancellotti P, Rodriguez Muñoz D (2016). ESC position paper on cancer treatments and cardiovascular toxicity developed under the auspices of the ESC committee for practice guidelines: the task force for cancer treatments and cardiovascular toxicity of the European society of cardiology (ESC). Eur Heart J.

[CR58] Zhong X, Li X, Qian L (2012). Glycine attenuates myocardial ischemia-reperfusion injury by inhibiting myocardial apoptosis in rats. J Biomed Res.

